# Semi-field evaluation of novel chemical lures for *Aedes aegypti*, *Culex quinquefasciatus*, and *Anopheles minimus* (Diptera: Culicidae) in Thailand

**DOI:** 10.1186/s13071-021-05108-3

**Published:** 2021-12-11

**Authors:** Dae-Yun Kim, Theerachart Leepasert, Michael J. Bangs, Theeraphap Chareonviriyaphap

**Affiliations:** 1 Department of Entomology, Faculty of Agriculture, Kasetsart University, Bangkok, Thailand; 2 Department of Chemistry, Faculty of Science, Kasetsart University, Bangkok, Thailand

**Keywords:** Attractant, BG-Lure, Kasetsart University-lure, BG-Sentinel trap, Black Hole ultraviolet light trap, Semi-field screen house assay

## Abstract

**Background:**

Entomological surveillance is an important means of assessing the efficacy of insect vector management programs and estimating disease transmission thresholds. Among baited traps, Biogents’ BG-Sentinel (BGS) trap baited with BG-Lure is considered to have the most similar outcome to, and be a possible replacement for, human-landing catches for the epidemiologically relevant monitoring of adult *Aedes aegypti* and *Culex quinquefasciatus*. In contrast to the BGS trap, the Black Hole ultraviolet (UV) light trap, which is widely used to catch nocturnal flying insects, is not baited with synthetic human odor-mimicking lures.

**Methods:**

We evaluated the l-lactic acid-based Kasetsart University (KU)-lures nos. 1–6 as novel candidate chemical lures for the diurnal species *Ae. aegypti* and the nocturnal species *Cx. quinquefasciatus *using two commercial traps (the BGS trap and the Black Hole UV light trap) in a semi-field screen (SFS) house. Firstly, we optimized the dose of each KU-lure in an SFS house (140 m^3^). Secondly, six different candidate KU-lures were screened by comparing their percent attraction using a single discriminating dose (0.5 g). Finally, we evaluated the synergism of the KU-lures selected in this way with commercially available traps.

**Results:**

BGS traps baited with KU-lure no. 1 exhibited the greatest percent attraction for *Ae. aegypti* (29.5% ± 14.3%), whereas those baited with KU-lure no. 6 most strongly attracted *Cx. quinquefasciatus* (33.3% ± 10.7%). Interestingly, BGS traps treated with 10 g BG-Lure did not significantly attract more *Ae. aegypti* or *Cx. quinquefasciatus* than the untreated BGS traps. CO_2_ at a flow rate of 250 ml/min most strongly attracted both *Ae. aegypti* and *Cx. quinquefasciatus* (42.2% ± 14.2% and 75.1% ± 16.9%, respectively). BGS and Black Hole UV light traps with KU-lure no. 6 exhibited a stronger attraction for *Cx. quinquefasciatus* than untreated traps, and the percent attraction did not differ between the treated traps.

**Conclusions:**

Synergistic effects of KU-lures nos. 1 and 6 with the mosquito traps were demonstrated for both the diurnal and nocturnal species in the SFS house assays. However, further studies are urgently needed for the development of species-specific lures to increase trap efficacy in the field for local vector mosquitoes in Thailand.

**Graphical Abstract:**

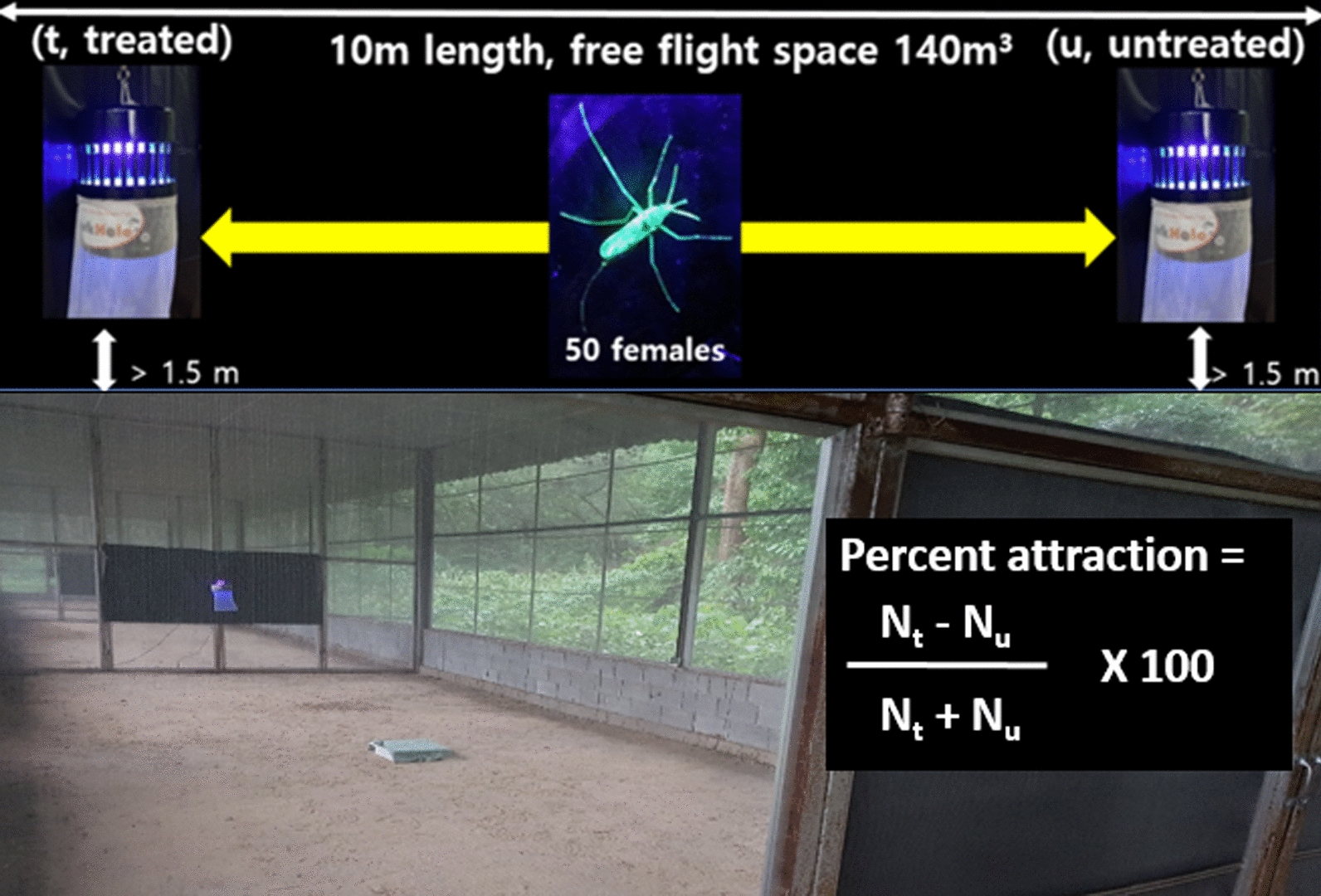

## Background

Dengue, the most prevalent vector-borne disease of public health importance, occurs in most countries in tropical and subtropical regions [1]. *Aedes aegypti* is the primary vector of dengue. This mosquito species exhibits highly anthropophilic feeding behavior, and is often found in close association with human dwellings [2, 3]. Typical larval habitats of this species include discarded tires filled with rainwater, flowerpots, empty oil drums, and large and small water storage containers [4]. Despite years of public health control activities and research progress on *Ae. aegypti* and dengue fever, there is still no commercially available effective and safe dengue vaccine. Prevention of dengue fever remains almost entirely reliant on the use of vector management and control practices, which are the most effective methods for reducing viral transmission in dengue-endemic areas [5]. Despite intensive efforts, the control of *Ae. aegypti* has proven extremely difficult because it is highly anthropophilic and thus found in close association with humans in domestic and peri-domestic environments. An accurate prediction of the abundance of *Ae. aegypti* females in a given area is important. One of the sampling techniques used to effectively assess and predict mosquito abundance is trapping [6].

Efforts have been made to develop better and more effective traps as integral components of surveillance systems for the monitoring and evaluation of vector control programs. Combinations of traps and lures have been successfully used for the control of several insect pests and vectors of disease [6, 7]. Devices which trap both adult and immature stages are important tools for the monitoring and surveillance of mosquito density [8, 9]. However, most traps are relatively ineffective for population sampling, and especially for adults of the day-biting *Ae. aegypti* [10, 11]. To overcome this critical limitation, several new traps have been developed and evaluated. The most promising of these include the BG-Sentinel (BGS) trap (Biogents, Regensburg, Germany). Several studies found that this trap efficiently captured more representative samples of adult *Ae. aegypti* populations than other traps [6, 7, 12].

Various chemical lures have been developed by using various types of olfactometers [13–20]. However, existing lures are not always effective in attracting *Ae. aegypti* from populations in different localities [21]. Therefore, lures should be developed using local field populations of species. In this study, we tested a laboratory-scale high-throughput screening system (HITSS) for the selection of candidate Kasetsart University (KU)-lures [22] for further testing in a semi-field screen (SFS) house using standard commercial mosquito traps, namely the BGS trap and the Black Hole ultraviolet (UV) light trap, for important vector mosquito species, including the diurnal *Ae. aegypti*, nocturnal *Culex quinquefasciatus*, and *Anopheles minimus*. In addition, traps with or without light sources were compared to assess their ability to trap *Cx. quinquefasciatus*. The results of the SFS house assay demonstrated the accuracy of the HITSS assay.

## Methods

### Mosquitoes

Larvae and pupae of *Ae. aegypti* were collected from natural or artificial breeding habitats in Pu Tuey Village, Kanchanaburi Province, Thailand (14°77′N, 99°11′E) in January 2019. This field population showed a high phenotypic resistance to 0.25% permethrin, with only 6% mortality, in the World Health Organization (WHO) tube bioassay [23]. Aquatic stages of *Cx. quinquefasciatus* were collected from sewage near a local restaurant at Thawi Watthana, Bangkok, Thailand (13°77′N, 100°34′E) in March 2020. The results of the WHO bioassay confirmed that permethrin resistance in the *Cx. quinquefasciatus* used here is moderate, with 60% mortality at 0.75% permethrin [22].

Field-collected individuals of both populations were immediately transferred to the insectary at the Department of Entomology, Faculty of Agriculture, Kasetsart University, Bangkok, Thailand to increase the size of the test populations. Aquatic stages (larvae and pupae) of the field populations were transferred to a field insectarium in Pu Teuy, Kanchanaburi Province, Thailand for the field study. The mosquitoes were reared under the same field insectary conditions (25 ± 5 °C, 80 ± 10% relative humidity, and 12-h:12-h light:dark photoperiod). Newly emerged F2 adults were provided with 10% cotton pads soaked with sugar solution. On each day of the study, newly emerged adults were transferred to a separate screen cage [(30 × 30 × 30 cm, length (L)  × width (W) × height (H)] so that the females’ ages could be determined. To increase the size of the population, sugar-fed F2 males and females were allowed to mate naturally in the screen cages for several days. Five-day-old females were blood-fed twice per week using a membrane feeding system with expired human blood obtained from the Thai Red Cross Society (Bangkok, Thailand). Two days post-blood-feeding, 10-cm-diameter oviposition dishes with (for *Ae. aegypti*) or without (for *Cx. quinquefasciatus*) moist filter paper were placed in the screen cages to collect the eggs.

Approximately 200–250 eggs were placed in individual plastic rearing trays [30 × 20 × 5 cm, L  × W × H] containing clean water. A few days later, granules of Optimum Nishikigoi Carp Fish Food (Perfect Companion, Samutprakarn, Thailand) were provided daily to feed the larvae of both species. Pupae were collected and transferred to the adult rearing cage covered with damp towels for adult emergence. Fifty nulliparous, 3- to 5-day-old sugar-starved females of each species were tested. At least 12 h before each test, the female mosquitoes were marked using fluorescent powder (BioQuip, Rancho Dominquez, CA) to distinguish them from the mosquitoes used in the previous assays and from wild mosquitoes that had accidentally flown in, following the method of Achee et al. [24]. If no dead or knocked-down mosquitoes were observed post-marking, the mosquitoes were considered healthy and used for testing.

Specimens of *An. minimus* were originally obtained from the Malaria Division, Department of Communicable Disease Control, Ministry of Public Health (Nonthaburi, Thailand) in 1998. This laboratory strain showed 100% mortality in response to permethrin exposure using the WHO bioassay [22]. The larvae were fed powdered Tetramin tropical fish food daily. The pupae were collected and transferred to a screen cage and allowed to develop into adults. The adults were provided with 10% sugar solution as an energy source. All of the mosquito cages were covered with damp towels to retain moisture. Non-blood-fed female mosquitoes (3–5 days old) were starved (provided only with water) for 24 h prior to testing.

### Experimental assays

#### Chemical lures

Four previously described [22] l-lactic-(+)-acid (Chemical Abstracts Service no. 79-33-4) based mixtures and two single compounds labeled as KU-lures (Table 1) were tested in an SFS house assay. A commercial product, namely the BG-Lure (Biogents), and CO_2_ supplied from a cylinder at a flow rate of 250 ml/min were used as the positive controls. The BG-Lure is available in a 10-g pack and consists of lactic acid, ammonia and caproic acid, which are used to mimic human sweat.

**Table 1:** Chemical components of Kasetsart University (KU) candidate lures

Candidate KU-lure	Chemical compound	Concentration^a^ (g/100 ml)
KU-lure no. 1	Lactic acid	10% w/v
	Octenol	2% w/v
	Isovaleric acid	4% w/v
KU-lure no. 2	Isoamyl alcohol	100% w/v
KU-lure no. 3	Octenol	100% w/v
KU-lure no. 4	Ammonium hydroxide	2.5% w/v
	Isovaleric acid	4% w/v
	Lactic acid	2% w/v
	Myristic acid	0.0025% w/v
KU-lure no. 5	Lactic acid	2% w/v
	Isovaleric acid	0.02% w/v
KU-lure no. 6	Lactic acid	2% w/v
	Octenol	0.25% w/v
	Isovaleric acid	0.5% w/v

*w*/*v* Weight/volume

^a^Distilled water was used as the solvent (Kim et al. [22])

#### BGS trap

**Fig. 1a–e:**
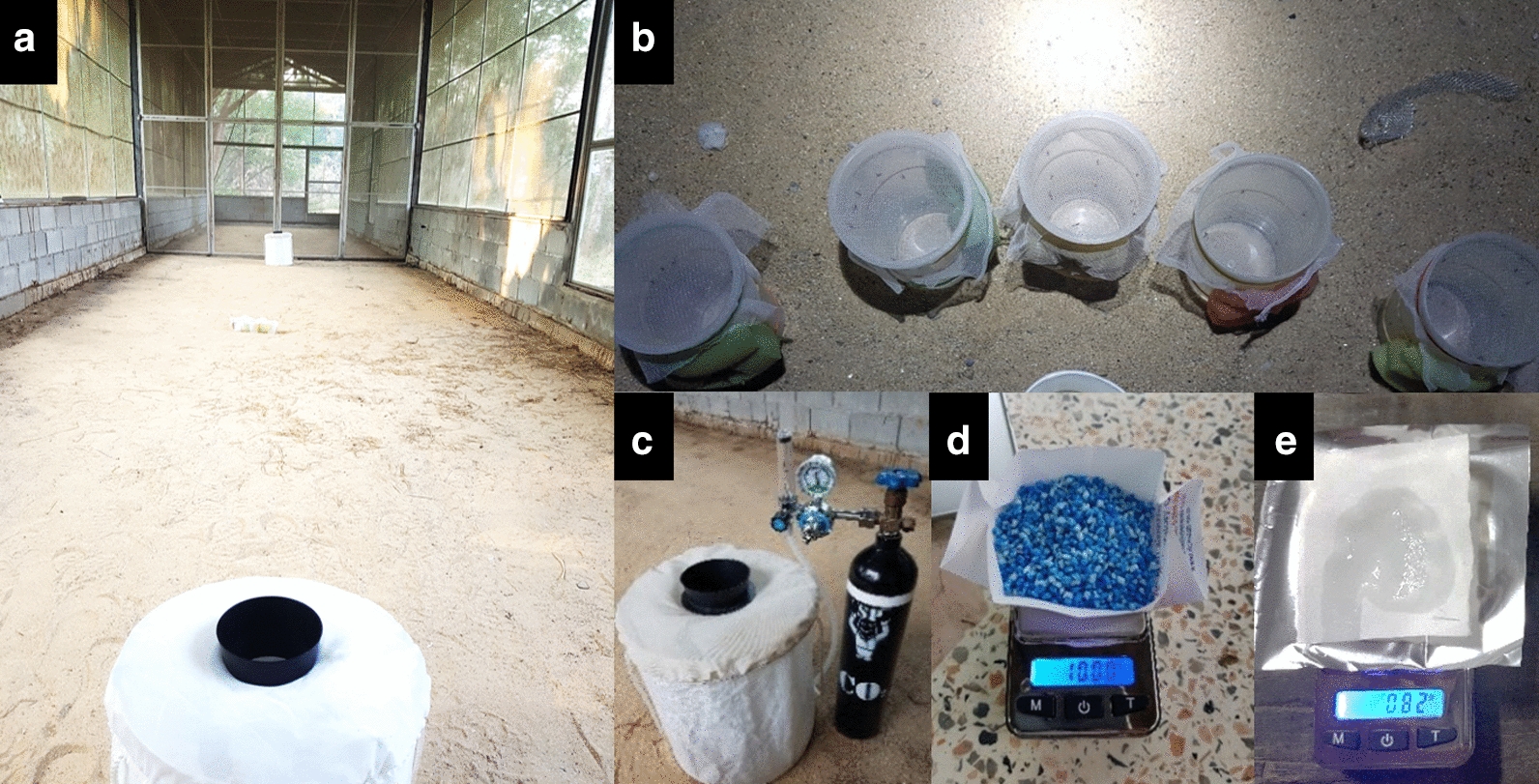
Semi-field screen (SFS) house assay set up. **a** Two Biogent BG-Sentinel (BGS) traps were placed 10 m apart. **b** Fifty females were released in the middle of the screen house. **c** CO_2_ tank. **d** BG lure. **e** Kasetsart University (KU) lure. Lures were placed inside the traps

Each tested lure was inserted into a BGS trap (Fig. 1) which was then placed on the ground at approximately 1 m from the wall to enable air to circulate around it. All the traps were operational for the entire experimental period. The diurnal species *Ae. aegypti* was tested during the daytime (0600–1800 hours), and the nocturnal species *Cx. quinquefasciatus* was tested during the nighttime (1800–0600 hours).

#### Black Hole UV light trap

**Fig. 2:**
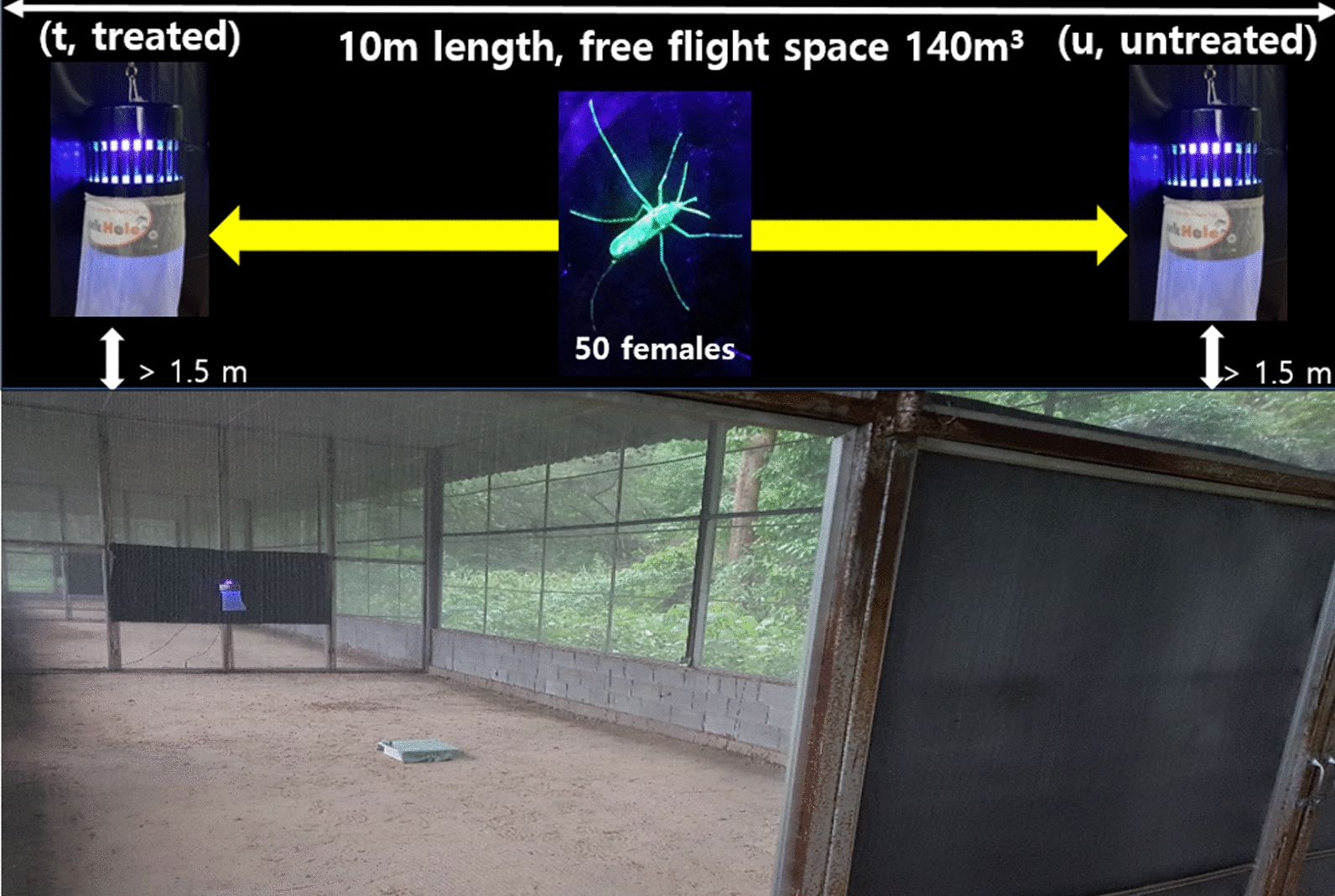
Black Hole ultraviolet (UV) light trap set up in semi-field screen (SFS) house assay

Two Black Hole UV light traps (manufactured from Bio-Trap Inc., Seoul, Korea, and purchased from Pan Science Co., Ltd., Bangkok, Thailand) were suspended 1.5 m above the ground from a chain attached to the top of each SFS cubicle (10 × 4 × 3.5 m, L × W × H] (Fig. 2). The total number of captured mosquitoes was determined after 12 h of collection by KU-lure no. 6-treated and untreated traps during the nighttime (1800–0600 hours).

#### SFS house assay

The screen house was subdivided into four 10-m-long cubicles separated by folding metal screen partitions. The volume of each cubicle was 140 m^3^ (4 m × 10 m × 3.5 m; Figs. 1–2), which approximated the volume of the existing SFS facility and the expected volume that *Ae. aegypti* would primarily use in and around a typical home in a dengue-endemic environment in Thailand. The environmental parameters temperature and relative humidity were measured for each separate cubicle using a hygrometer (Yuequing Xinyang Technology, Yuequing City, Zhejiang Province, China), and did not significantly differ between them (Table 2). This study was conducted from February to July in 2019 and in 2020.

**Table 2:** Temperature and relative humidity (mean ± SD) of each semi-field screen (SFS) house cubicle during the daytime and nighttime, May 2019, Pu-Teuy Village, Kanchanaburi Province, Thailand

Time period	Cubicle	Temperature (°C) (mean ± SD)	Relative humidity (%) (mean ± SD)
Day (0600–1800 hours)	1	29.1 ± 3.9 a	78.2 ± 17.7 a
	2	29.0 ± 3.8 a	78.4 ± 17.4 a
	3	29.9 ± 3.6 a	73.4 ± 13.5 a
	4	27.2 ± 3.1 a	78.3 ± 10.7 a
Night (1800–0600 hours)	1	25.0 ± 1.9 a	99.0 ± 0.0 a
	2	25.0 ± 1.5 a	99.0 ± 0.0 a
	3	24.7 ± 2.5 a	93.6 ± 1.2 a
	4	24.5 ± 1.6 a	99.0 ± 0.0 a

For each parameter in each time period, the same lowercase letters indicate no significant difference between cubicles (day, ANOVA; nighttime, Kruskal–Wallis *H*-test; 95% confidence limits)

We optimized the dose of the previously selected [22] KU-lure no. 1 (for *Ae. aegypti*) and KU-lure no. 6 (for *Cx. quinquefasciatus*) by testing them at five different concentrations in the SFS house cubicles (Table 3). Then, to test the accuracy of the HITSS assay, six different KU-lures at the optimized dose were screened in a SFS house, which was 50,000 times larger in volume than the space used in the laboratory-scale assay (2.75 L). In addition, the percent attraction of lure-treated and untreated traps was compared to assess synergism.

**Table 3:** Responses of adult female *Aedes aegypti* and *Culex quinquefasciatus* to Biogents’ BG Sentinel (BGS) traps equipped with different amounts of KU-lures in the SFS house assay

Species	Lures	Amounts (g)	No. of mosquitoes caught in BGS traps (mean ± SD)		*P*^a^	Capture (%) (mean ± SD)	Attraction^b^ (%) (mean ± SD)
			Untreated	Treated			
*Ae. aegypti*	KU no. 1	0.0	22.3 ± 2.1	22.9 ± 4.1	0.705	90.3 ± 8.7 a	0.8 ± 11.2 b
		0.1	17.3 ± 3.0	26.1 ± 4.2	0.000*	86.8 ± 9.4 a	20.2 ± 12.9 ab
		0.5	15.1 ± 2.9	28.1 ± 4.7	0.000*	86.5 ± 7.7 a	29.5 ± 14.3 a
		1.0	30.6 ± 5.2	16.9 ± 3.9	0.000	95.0 ± 8.1 a	− 28.5 ± 17.5 c
		1.5	30.4 ± 4.9	11.0 ± 2.9	0.000	82.8 ± 10.3 a	− 46.7 ± 12.8 cd
		2.0	37.8 ± 3.5	9.6 ± 3.2	0.000	94.8 ± 8.4 a	− 59.8 ± 11.6 d
*Cx. quinquefasciatus*	KU no. 6	0.0	16.0 ± 7.3	14.0 ± 5.5	0.546	60.0 ± 18.4 a	− 5.0 ± 29.4 b
		0.1	16.1 ± 1.6	22.0 ± 6.9	0.035*	76.3 ± 14.2 a	12.6 ± 21.5 ab
		0.5	10.5 ± 3.8	20.6 ± 5.3	0.001*	62.3 ± 17.3 a	33.3 ± 10.7 a
		1.0	18.8 ± 9.3	10.1 ± 4.9	0.041	57.8 ± 28.3 a	− 29.6 ± 9.9 cd
		1.5	17.9 ± 8.9	10.9 ± 6.6	0.095	57.5 ± 28.0 a	− 28.7 ± 25.6 cd
		2.0	31.4 ± 4.9	9.9 ± 2.9	0.000	82.5 ± 10.0 a	− 51.9 ± 13.6 d

Eight replicates per species (50 females per replicate) were tested (*n* = 400). For each species, different lowercase letters within a column indicate significant difference according to one-way ANOVA with post hoc Tukey’s honest significant difference test when *P* < 0.05. For other abbreviations, see Tables 1 and 2

*P < 0.05 (significantly more females in the treated BGS trap)

^a^Student’s *t*-test between untreated and treated traps (*P* < 0.05)

^b^Percent attraction = (no. of mosquitoes in treated traps – no. of mosquitoes in untreated traps)/(no. of mosquitoes in treated traps + no. of mosquitoes in untreated traps) × 100

The screen house and traps were regularly cleaned to remove predators that otherwise may have consumed the trapped mosquitoes. Fifty marked healthy females were released in the middle of a single SFS room in which two BGS traps had been placed for *Ae. aegypti* and for *Cx. quinquefasciatus* (Fig. 1) or two Black Hole UV light traps had been placed for *Cx. quinquefasciatus* and *An. minimus* (Fig. 2) [25]. Eight (*n* = 400) and nine (*n* = 450) replicates were used for the BGS traps and Black Hole UV light traps, respectively.

### Statistical analysis

Environmental parameters (temperature and relative humidity) data are presented as the mean ± SD for four SFS cubicles as determined using the Kruskal–Wallis *H*-test for multiple comparisons for nighttime (*P* < 0.05). Data for daytime were subjected to one-way ANOVA, and means were compared using Tukey’s honest significant difference test at 95% confidence level. The numbers of mosquitoes in lure-treated and untreated traps were compared using Student’s *t*–test to determine statistically significant differences. The percent attraction was calculated from the obtained values using following formula:

$${\text{Percent attraction}}\, = \,\left( {N_{{\text{t}}} {-}N_{{\text{u}}} } \right)/\left( {N_{t} \, + \,N_{u} } \right)\, \times \,{1}00$$

where *N*_t_ is the number of mosquitoes captured in baited traps and *N*_u_ is the number of mosquitoes in untreated traps. If the tested lure is attractive, the percent attraction is positive. Conversely, a negative value indicates that the lure is not attractive. The species-specific percent attraction of the tested lures was compared using ANOVA. All the statistical analyses were performed with SPSS version 28 (IBM, Armonk, NY). Statistical significance is indicated by *P* < 0.05.

## Results

### Optimization assay

Discriminating doses for the SFS house assay were selected for *Ae. aegypti* and *Cx. quinquefasciatus.* Five different amounts of KU-lures nos. 1 and 6 (0.1, 0.5, 1.0, 1.5, 2.0 g) and no lure (0 g) were evaluated for each species of mosquito (Table 3). The BGS traps baited with 0.1 g or 0.5 g of KU-lure no. 1 captured significantly more *Ae. aegypti* than untreated BGS traps [0.1 g, *t*_(14)_ = −4.891, *P* = 0.000; 0.5 g, *t*_(14)_ = −6.654, *P* = 0.000]. Specifically, BGS traps baited with 0.5 g of KU-lure no. 1 had the highest percent attraction (29.5% ± 14.3%). When no KU-lure was used (control, 0 g), the number of captured *Ae. aegypti* mosquitoes did not significantly differ between the BGS traps [22.3 ± 2.1 vs 22.9 ± 4.1; *t*_(14)_ = −0.387, *P* = 0.705]. BGS traps treated with 1.0 g, 1.5 g or 2.0 g of KU-lure no. 1 captured significantly fewer *Ae. aegypti* than untreated traps [1.0 g, *t*_(14)_ = 5.933, *P* = 0.000; 1.5 g, *t*_(14)_ = 9.605, *P* = 0.000; 2.0 g, *t*_(14)_ = 16.995, *P* = 0.000]. The results were similar for *Cx. quinquefasciatus *with KU-lure no. 6, as baiting the traps with 0.1 g or 0.5 g of this lure significantly increased the number of mosquitoes captured [0.1 g, *t*_(14)_ = −2.327, *P* = 0.035; 0.5 g, *t*_(14)_ = −4.390, *P* = 0.001]. From the results, we selected 0.5 g as the discriminating dose for the 140-m^3^ SFS house for all candidate KU-lures and tested species. The optimization assay indicated that there was no significant difference among the cubicles for either mean temperature or relative humidity (Table 2).

### Screening assay

BGS traps baited with standard BG-Lure, CO_2_ or KU-lures were evaluated to compare their percent attraction for *Ae. aegypti* and *Cx. quinquefasciatus* in the SFS system. The standard BG-Lure at 10 g did not attract either *Ae. aegypti* or *Cx. quinquefasciatus*. CO_2_ at a flow rate of 250 ml/min exhibited the greatest percent attraction for both species. BGS traps baited with CO_2_ had a significantly higher percent attraction [*t*_(14)_ = 4.218, *P* = 0.001] for *Cx. quinquefasciatus* (75.1% ± 16.9%) than for *Ae. aegypti* (42.2% ± 14.2%). There was no significant difference [*t*_(14)_ = 0.591, *P* = 0.564] in the high attractancy of BGS traps baited with 0.5 g of KU-lure no. 6 for *Cx. quinquefasciatus* (33.3% ± 10.7%) and those baited with 0.5 g of KU-lure no. 1 for *Ae. aegypti* (29.5% ± 14.3%). The other candidate KU-lures did not attract mosquitoes, with the exception of KU-lure no. 4, which had a positive percent attraction for *Cx. quinquefasciatus*, albeit this was not statistically significant relative to the control [*t*_(14)_ = 1.003, *P* = 0.333] (Table 4).

**Table 4:** Responses of adult female *Aedes aegypti* and *Culex quinquefasciatus* to BGS traps equipped with different candidate KU-lures in the SFS house assay

Species	Lure	No. of mosquitoes caught in BGS traps (mean ± SD)		*P*^a^	Capture (%) (mean ± SD)	Attraction^b^ (%) (mean ± SD)
		Untreated	Treated			
*Ae. aegypti*	KU no. 1	15.1 ± 2.9	28.1 ± 4.7	0.000*	86.5 ± 7.7 a	29.5 ± 14.3 ab
	KU no. 2	34.0 ± 4.2	11.6 ± 3.5	0.000	91.3 ± 4.5 a	− 48.9 ± 15.6 e
	KU no. 3	29.1 ± 8.6	13.5 ± 4.9	0.001	85.3 ± 21.6 a	− 36.3 ± 15.3 de
	KU no. 4	36.8 ± 4.3	12.9 ± 3.6	0.000	99.3 ± 6.8 a	− 48.1 ± 14.4 e
	KU no. 5	31.8 ± 6.1	12.1 ± 5.8	0.000	87.8 ± 6.3 a	− 44.8 ± 25.8 e
	KU no. 6	23.4 ± 7.0	20.8 ± 7.1	0.469	88.3 ± 5.4 a	− 6.1 ± 30.6 cd
	BG-Lure	23.3 ± 6.3	21.3 ± 4.6	0.482	89.0 ± 11.5 a	− 3.6 ± 21.1 c
	CO_2_	14.1 ± 3.3	34.9 ± 4.0	0.000*	98.0 ± 2.4 a	42.2 ± 14.2 a
	Control	22.3 ± 2.1	22.9 ± 4.1	0.705	90.3 ± 8.7 a	0.8 ± 11.2 bc
*Cx. quinquefasciatus*	KU no. 1	34.6 ± 4.4	8.3 ± 1.0	0.000	85.8 ± 6.9 a	− 61.0 ± 7.1 f
	KU no. 2	28.6 ± 4.7	13.4 ± 6.1	0.000	84.0 ± 9.4 a	− 37.4 ± 25.3 def
	KU no. 3	24.5 ± 6.3	16.8 ± 3.8	0.010	82.5 ± 12.6 ab	− 17.7 ± 19.0 cde
	KU no. 4	18.8 ± 5.3	22.1 ± 5.2	0.223	81.8 ± 7.3 ab	8.6 ± 24.4 bc
	KU no. 5	27.0 ± 5.2	11.3 ± 3.5	0.000	76.5 ± 12.7 abc	− 41.5 ± 14.7 ef
	KU no. 6	10.5 ± 3.8	20.6 ± 5.3	0.001*	62.3 ± 17.3 bc	33.3 ± 10.7 b
	BG-Lure	20.0 ± 2.8	18.0 ± 5.1	0.350	76.0 ± 8.2 abc	− 6.5 ± 19.9 cd
	CO_2_	4.6 ± 2.7	34.3 ± 8.3	0.000*	77.8 ± 15.2 abc	75.1 ± 16.9 a
	Control	16.0 ± 7.3	14.0 ± 5.5	0.546	60.0 ± 18.4 c	− 5.0 ± 29.4 c

Eight replicates (50 females per replicate) were tested (*n* = 400). Each candidate KU-lure was used at the discriminating dose (0.5 g). The positive controls were CO_2_ at 250 ml/min and the BG-Lure at 10 g (contents of a single pack). For each species, different letters within columns indicate significant difference by one-way ANOVA with post hoc Tukey’s honest significant difference test when *P* < 0.05. For abbreviations, see Tables 1, 2 and 3

*P < 0.05 (significantly more females in the treated BGS trap)

^a^Student’s *t*-test between untreated and treated traps (*P* < 0.05)

^b^Percent attraction = (no. of mosquitoes in treated trap − no. of mosquitoes in untreated trap)/(no. of mosquitoes in treated trap + no. of mosquitoes in untreated trap) × 100

### Synergism assay

The Black Hole UV light trap treated with the optimized KU-lure no. 6 was evaluated to compare the percent attraction for *Cx. quinquefasciatus* and *An. minimus* to that of untreated traps in an SFS house assay. The two species were captured similarly by the UV light trap without lures [*Cx. quinquefasciatus*, *t*_(16)_ = 0.585, *P* = 0.567; *An. minimus*, *t*_(16)_ = 0.333, *P* = 0.743]; the respective capture rates were 74.0% ± 13.2% and 76.4% ± 13.0%. Significantly more *Cx. quinquefasciatus* mosquitoes were captured (24.9 ± 6.1) when the trap was baited with 0.5 g of KU-lure no. 6 than when the trap was not baited (15.6 ± 5.4) [*t*_(16)_ = −3.424, *P* = 0.003]. Although more *An. minimus* were captured in traps baited with KU-lure no. 6 (23.8 ± 8.7) than in the control (18.9 ± 9.6), the difference was not statistically significant [*t*_(16)_ = −1.134, *P* = 0.274]. The capture rates increased for *Cx. quinquefasciatus* and for *An. minimus* with KU-lure no. 6, to 80.9% ± 10.2% and 85.3% ± 6.6%, respectively, but the differences were not statistically significant. Overall, although the percent attraction for both species was positive (22.8 ± 25.1% for *Cx. quinquefasciatus* and 12.4 ± 43.5% for *An. minimus*), significant result was only found from *Cx. quinquefasciatus* with the optimized and screened KU-lure no. 6 treated Black Hole UV light trap compared to untreated trap (Table 5).

**Table 5:** Responses of *Culex quinquefasciatus* and *Anopheles minimus* to Black Hole ultraviolet (UV) light traps equipped with KU-lure no. 6 in the SFS house during nighttime

Species	Lure	Amounts (g)	No. of mosquitoes in Black Hole traps (mean ± SD)		*P*^a^	Capture (%) (mean ± SD)	Attraction^b^ (%) (mean ± SD)
			Untreated	Treated			
*Cx. quinquefasciatus*	KU no. 6	0.5	15.6 ± 5.4	24.9 ± 6.1	0.003*	80.9 ± 10.2 a	22.8 ± 25.1 a
	Control	0.0	19.3 ± 5.6	17.7 ± 6.5	0.567	74.0 ± 13.2 a	− 5.0 ± 27.7 b
*An. minimus*	KU no. 6	0.5	18.9 ± 9.6	23.8 ± 8.7	0.274	85.3 ± 6.6 a	12.4 ± 43.5 a
	Control	0.0	19.6 ± 5.5	18.7 ± 5.9	0.743	76.4 ± 13.0 a	− 3.0 ± 21.7 a

Nine replicates (50 females per replicate) were tested (*n* = 450). For each species, different letters within the same column indicate significant difference by Student’s *t*-test when *P* < 0.05. For other abbreviations, see Tables 1 and 2

*P < 0.05 (significantly more females in the treated Black Hole UV light trap)

^a^Student’s *t*-test between untreated and treated traps (*P* < 0.05)

^b^Percent attraction = (no. mosquitoes in treated trap − no. mosquitoes in untreated trap)/(no. mosquitoes in treated trap + no. mosquitoes in untreated trap) × 100

## Discussion

This aim of this study was to optimize lures used to attract mosquitoes for their possible use as integral components of surveillance systems for the monitoring and evaluation of vector control programs. Trap and lure combinations were tested in an SFS house trial with the aim of improving future mosquito surveillance. Trap and lure combinations have been successfully applied to the control of several insect taxa, including mosquitoes [26] and tsetse flies [27]. Although traps are important tools for the surveillance of mosquito abundance [9], most of them are relatively ineffective, and especially so for day-biting mosquitoes such as *Ae. aegypti* [10, 28].

The BGS trap has been suggested as a potential replacement for human-landing catches in the case of *Ae. aegypti* [7]. However, the BGS trap baited with the BG-Lure is expensive and, as it is licensed in the USA, cannot be manufactured in lower-income countries, some of which have dengue rates of up to 75%, in particular in Southeast Asia. There are various types of lower priced traps on the market, most of which use UV light-emitting diode (LED) light sources**.** However, there is little documented scientific evidence of the efficacy of these types of traps. In addition, populations of a mosquito species may respond differently according to their geographical location. To overcome these problems, the local development of lures using field-collected mosquitoes is necessary. In particular, economical and simple methodologies are critical for the development of mosquito attractants in less well-equipped laboratories. Recently, Kim et al. [22, 23] evaluated a potential tool for lure development, the HITSS assay. However, more data are needed to demonstrate its accuracy in larger-scale SFS house set ups.

In the present study, the results of the BGS trap baited with an appropriate amount of the KU-lures (0.5 g) were extremely promising, as they were similar to those of other lure-baited traps. Although CO_2_ alone was the strongest attractant for *Ae. aegypti*, the results were not significantly different from those when traps were baited with 0.5 g of KU-lure no. 1. CO_2_ at the appropriate concentration is considered the strongest cue for trapping mosquitoes [29]. However, inappropriate concentrations of CO_2_ repel female mosquitoes [30, 31]. Although *Ae. aegypti* was attracted to CO_2_ at the appropriate concentration, the percent attraction for this species was significantly lower than that for *Cx. quinquefasciatus*. This difference was also seen in a previous study using the HITSS assay, where *Cx. quinquefasciatus* was more strongly attracted by 0.1 g of dry ice than *Ae. aegypti *[23]. We also found that the amounts of the lure ingredients were among the most crucial factors for mosquito attraction and repellence. For example, KU-lures no. 1 and no. 6, and BG-Lure, contain the common component, lactic acid, at different proportions (10%, 2%, and 20%–40%, respectively). However, the KU-lures contain lactic acid as a primary component at higher proportions than the BG-Lure. In addition to lactic acid, the BG-Lure contains other important mosquito attractants such as ammonium hydrogen carbonate and hexanoic acid along with inert ingredients in different proportions. Therefore, the response of mosquitoes to these lures varies according to their chemical components and the proportions of these.

Octenol, the other key component of KU-lures, is present in human breath and sweat, and is known to play a significant role in mosquito responses to human hosts [32]. However, pure (100%) octenol alone does not attract mosquitoes, and Salazar et al. [33] found that octenol works synergistically with CO_2_ to attract *An. gambiae* and *Ae. aegypti* (anthropophilic species) but not *Cx. quinquefasciatus*, which prefers non-human hosts (it is ornithophilic) that do not emit octenol [34–36]. A small amount of octenol (0.25%) exerted a synergetic effect when mixed with 2% lactic acid (KU-lure no. 6) on *Cx. quinquefasciatus* attraction, whereas *Ae. aegypti* was attracted to mixtures containing higher amounts of lactic acid (10%) and octenol (2%). These results strongly indicate that the most effective species-specific attractants are mixtures (as opposed to single compounds) that contain compounds at different proportions and ratios. Therefore, to develop the best chemical lure for different populations of species, multiple candidate lures should be evaluated using a simple olfactometer in the laboratory, and the results confirmed by large-scale SFS house assays.

Mosquitoes can detect specific odors that stimulate various behaviors (e.g., nectar-seeking, mating, host-seeking, oviposition) from among thousands of different chemicals. Olfactory receptors, located in hair-like sensory organs (sensilla) on antennae and palps, participate in host-seeking behaviors [37]. In general, there is a single olfactory receptor neuron for each odor molecule [38–41]. However, some olfactory receptor neurons respond to several odor compounds, and thus may elicit a stronger attraction to a mixture than to a single compound [42]. The lure development process should include a large-scale SFS house assay to determine the actual efficacy of potential candidate lures developed in the laboratory. SFS house assay results could guide developers with regard to adjustments to the composition of potential candidate lures to increase their attractiveness. In other words, the process of lure development requires both SFS and laboratory assays to assess and increase the attractancy of lures for specific species. Additionally, the use of local mosquito populations for this is critical, as different populations and also strains of a mosquito species may display diverse response patterns. It was also confirmed in this study that the sensitivity of the diurnal and nocturnal species to the lures significantly differed. Although the diurnal species was attracted to visual cues (e.g., high contrast), the nocturnal species may have been more sensitive to chemical cues in the dark. We confirmed that both in the presence (Black Hole) and absence (BGS) of a light source, lure treatment increased the attractancy of both traps. However, the light source also attracted flying insects other than mosquitoes, such as beetles, moths, and fireflies, which can influence trap efficacy and the accuracy of entomological surveillance.

Effective mosquito surveillance tools combined with a chemical lure can be used as key components of a push–pull system along with a spatial repellent near residential areas. Salaza et al. [25] confirmed that there was no significant difference in the capture rate of BGS traps surrounding experimental huts between mosquitoes that had been previously exposed to the spatial repellent and those that had not. Furthermore, no meaningful relation between pesticide resistance and host-seeking behavior could be confirmed in laboratory experiments using the HITSS assay [22, 23]. However, further study of the persistence of the chemical lures in the material to which they are applied should be considered. Also, the performance of the lures should be compared when human hosts are present in residential buildings to assess the epidemiological effectiveness of these vector control measures.

Trap location and density are important factors for trapping efficacy [20]. Salazar et al. [25] examined trap density using different numbers of mosquitoes in a SFS. They confirmed that two traps were a suitable number for each SFS cubicle regardless of mosquito density in a range of 10–250 females. In addition, they found that the traps had the highest impact (impact period) in the first few hours. In this study, we observed a similar phenomenon for *Ae. aegypti* active between 0600 and 0900 hours (data not shown).

During our SFS experiments, which were conducted from February to July, the lower temperature range was 24.2–25.9 °C and the higher one 33.9–37.3 °C. However, on average, in the morning (0600–0900 hours) during the whole study period, the temperature and relative humidity were consistently lower than 30 °C and nearly 90%, respectively. As the mosquitoes tested here are most active at around 25 °C, and prefer high humidity [10, 16, 43], the number of mosquitoes captured during periods of high temperature (over 33 °C) and low relative humidity (less than 55%) dramatically reduced in the SFS facility at 1000–1500 hours. These patterns are in agreement with those of previous studies on the peak period of host-seeking in mosquitoes when a human landing collection method is used [44–46]. In short, mosquitoes search for hosts by detecting multiple cues, which can be visual, mechanical, or chemical [47, 48]. Environmental factors such as air temperature, relative humidity and air movement also affect a female mosquito’s host-seeking behavior [49]. For instance, it was found that when air had a higher moisture content and was warm, significantly more mosquitoes were attracted by CO_2_ [43].

Semi-field-scale screen houses allow researchers to determine a selected lure’s effectiveness for target species under free-flying conditions [19]. Recent WHO guidelines for the development of trapping methods for mosquitoes [20] state that the benefits of using screen-enclosed facilities are that results can be determined for exact numbers of mosquitoes of known ages and species-specific behavior recorded under local natural conditions of temperature, light, humidity and air movement, all of which are measurable.

In this study, synergistic effects between selected novel KU-lures and commercial traps were successfully demonstrated in the SFS house. However, the numbers of wild nocturnal mosquitoes captured by the lure-treated and untreated Black Hole UV light traps were not significantly different when we conducted field trials in experimental huts located in Pu-Teuy Village, Kanchanaburi Province in July 2021 (data not shown). These results are similar to those for the lure-treated and untreated BGS traps during the daytime. Furthermore, in terms of the number of mosquitoes trapped during nighttime, the BGS traps with KU-lures could not beat either the lure-treated or untreated Black Hole UV light trap. It is thus clear that a UV light source is a powerful attractant for the nocturnal species *Cx. quinquefasciatus* (Kim et al., unpublished data).

Recent studies that evaluated the efficacy of multiple low-cost light traps revealed that light of different colors, wavelengths and types of UV light sources (e.g., fluorescent or LED) directly affect trap performance [50, 51]. Both wavelength and light intensity are important factors for mosquito traps [52]. Fluorescent lights and LEDs differ in the range of wavelengths that they emit, with the former emitting a broad range and the latter a narrow one, although these can be adjusted by changing the diode settings from lower to higher wavelengths [53]. A recent study, conducted in an urban area of Bangkok, Thailand which used Black Hole traps [51] with multiple colored LED and fluorescent lamps, showed that the UV fluorescent lamp attracted the highest number of nocturnal mosquitoes; in addition, the selected LED UV-A wavelengths (315–400 nm) had an outstanding effect elsewhere [54, 55]. LEDs are cost-effective because they have a longer lifespan and are also less fragile than fluorescent glass lamps, which need to be replaced more frequently [56]. UV LED lamps are considered a sustainable alternative to high-energy fluorescent lamps, which are actually no longer available for sale in some countries to help cut emissions and increase energy saving. In England, for example, it is predicted that LEDs will account for 85% of total sales of light sources by 2030; this should lead to a reduction of 1.26 million tons of emitted carbon, which is equal to half the CO_2_ emissions of a million cars. As various types of low-cost UV LED light traps are commercially available [50], further studies of the synergistic effects of various wavelength ranges with combinations of chemical lures are urgently needed to increase the efficacy of these affordable and sustainable tools so that they can be effectively used in the entomological surveillance of national vector management programs.

## Conclusions

The SFS house assay used here effectively demonstrated the synergism of traps with KU-lures for the attraction of both diurnal and nocturnal mosquito species. However, further research is urgently needed for the development of species-specific lures to increase trap efficacy.

## Data Availability

The datasets supporting the conclusions of this article are included within the article. Raw data are available from the corresponding author on reasonable request.

## Abbreviations

BGS: BG-Sentinel
KU: Kasetsart University
SFS: Semi-field screen
WHO: World Health Organization
